# Characterisation of psoriasis susceptibility locus 6 (PSORS6) in patients with early onset psoriasis and evidence for interaction with PSORS1

**DOI:** 10.1136/jmg.2008.065029

**Published:** 2009-06-11

**Authors:** U Hüffmeier, J Lascorz, T Becker, F Schürmeier-Horst, A Magener, A B Ekici, S Endele, C T Thiel, S Thoma-Uszynski, R Mössner, K Reich, W Kurrat, T F Wienker, H Traupe, A Reis

**Affiliations:** 1 Institute of Human Genetics, University Hospital Erlangen, University Erlangen-Nuremberg, Germany; 2 Institute of Medical Biometry, Informatics and Epidemiology, University of Bonn, Germany; 3 Department of Dermatology, University of Münster, Germany; 4 Department of Pathology, University of Erlangen, Germany; 5 Department of Dermatology, University of Erlangen, Germany; 6 Department of Dermatology, University of Göttingen, Germany; 7 Dermatologikum Hamburg, Hamburg, Germany; 8 Asklepios Nordseeklinik, Westerland/Sylt, Germany

## Abstract

**Background:**

Psoriasis is a genetically complex, chronic inflammatory skin disease. The authors have previously identified a susceptibility locus on chromosome 19p13 (PSORS6).

**Methods and results:**

In a follow-up linkage disequilibrium (LD) study in an independent family based cohort, the authors found evidence for association to a newly discovered microsatellite at this locus (D19SPS21, p<5.3×10^−5^). An LD based association scan in 300 trios revealed association to several single, single nucleotide polymorphisms (SNPs) in one LD block. When the authors stratified this cohort for carrying the PSORS1 risk allele at the *HLA-C* locus, evidence for association became much stronger at single SNP and haplotype levels (p values between 1.0×10^−4^ and 8.0×10^−4^). In a replication study of 1114 patients and 937 control individuals, evidence for association was also observed after stratification to the PSORS1 risk allele. In both study groups, logistic regression showed evidence for interaction between the risk alleles at PSORS1 and PSORS6. Best p values for rs12459358 in both study groups remained significant after correction for multiple testing. The associated LD block did not comprise any known genes. Interestingly, an adjacent gene, *MUC16*, coding for a large glycosylated protein expressed in epithelia and of unknown function, could be shown to be also expressed in tissues relevant for pathogenesis of psoriasis such as skin and thymus. Immunohistochemical analyses of skin revealed focal staining for MUC16 in suprabasal epidermal cells. Further functional studies are required to clarify its potential role in psoriasis and identify the causal variant(s) at this locus.

**Conclusion:**

The data establish PSORS6 as a confirmed psoriasis susceptibility locus showing interaction with PSORS1.

Psoriasis is a chronic inflammatory disorder of the skin. The disease has a complex aetiology and affects about 0.5–4.0% of the European and Northern American population.^1^ ^2^ Even though there is considerable evidence for genetic factors from family and twin studies—for example, concordance rate of up to 65% in monozygotic twins—only a few susceptibility factors have been discovered to date. While positional cloning strategies using genome wide linkage analysis (GWLA) have allowed identification of disease causing variants in numerous monogenic diseases, the adaptation of this systematic approach to complex diseases^3^ has been successful in only a few instances. In psoriasis vulgaris (PsV), the most evidentiary linkage region is at psoriasis susceptibility locus 1 ( =  PSORS1) on chromosome 6p21.3,^4^ especially in families with PsV manifesting at younger age (⩽40 years, type I psoriasis^5^). At this locus, the most consistently associated allele is the *Cw0602 allele of *HLA-C* gene, although the remarkably high linkage disequilibrium (LD) at this locus has hampered definite identification of the causative variant.^6^ In addition, more than nine psoriasis susceptibility loci have been identified (PSORS1-9 and one further for psoriatic arthritis, PSORAS1). Only at three loci, replicated association to candidate genes (*RAPTOR* and *SLC12A8*) has been reported so far (PSORS2 and PSORS5)^7^ ^8^ ^9^ ^10^ or several genes—for example, *LOR*, *LCE1C*, *PGLYRP*, *SPRR* genes, *PRR9* genes and *IVL*—have been proposed to account for psoriasis susceptibility (PSORS4).^11^ ^12^ ^13^ ^14^ In contrast, genome-wide association studies (GWAS) have turned out to be a very successful approach for many complex diseases. Recently, variants in two genes of the IL-23R pathway were identified as PsV susceptibility factors by two independent groups.^15^ ^16^ Like in other HLA related complex traits, the relative risk associated with these variants is markedly lower than that for *HLA-C* associated allele. It is expected that many more susceptibility alleles with small effects contribute to the aetiology of PsV.

In this study, we describe our positional cloning efforts at PSORS6 located on chromosome 19p13, which we previously identified in a genome-wide linkage scan of extended multiplex PsV families from Germany.^17^ Subsequently, we detected genetic association in an independent family based cohort to two newly discovered microsatellite markers below the linkage peak. After exploring the LD structure at this previously poorly characterised genomic region, we performed association studies based on the LD structure and could narrow a PsV susceptibility allele to a 50 kb intergenic LD block. This finding was replicated in a further case–control cohort. In both groups, association was stronger in patients carrying the PSORS1 risk allele, suggesting interaction between both loci. Strong LD hampered further refinement of the locus, which contains no known gene. One of the neighbouring genes (*MUC16*), though, showed expression in several tissues relevant for PsV and the product of this gene showed immunostaining in epidermal cells. We speculate that this locus contains yet unidentified regulatory elements influencing other genes in this genomic region.

## Methods

### Patients

The 210 nuclear families with and without 90 additional trios, respectively, were the ones described previously.^18^ ^19^ In brief, all index cases had an early onset form of psoriasis vulgaris with medium (SD) age of onset of 16.2 (9.1) years; 53.3% of index patients were male. In 61% of trios, the father and/or the mother were also affected by psoriasis.

The case cohort consisted of 1114 single patients with PsV and was recruited through dermatology clinics at three psoriasis rehabilitation hospitals and at three university hospitals.^20^ An early onset form of psoriasis (type I) was diagnosed in all but 75 patients. The medium age of onset was 23.2 (11.9) years, and 21.4 (9.5) years in type I patients. The majority of patients suffered from plaque type of PsV. We excluded all patients with signs of psoriatic arthritis until the time of recruitment when medium age was 48.2 (13.4) years; 62% of patients were male.

The 937 control probands had no PsV and no history or signs of inflammatory joint disease at the time of recruitment, when their mean age was 31.6 (10) years. All were German (white) healthy blood donors; 58% of probands were male.

The studies were approved by the ethical committees of the University of Erlangen-Nuremberg and of the University of Münster. Written informed consent was obtained from each patient and control proband before enrolment. The investigations were conducted according to Declaration of Helsinki principles.

### Microsatellite analyses

To identify new microsatellites within the linkage region, we used the program Tandem Repeat Finder (Benson^21^; http://tandem.bu.edu/trf/trf.html (accessed April 2002)). In order to have markers with fewer alleles, we chose a set of 24 evenly spaced microsatellites that were mostly tetra- or penta-nucleotides or had in the case of di-nucleotides <15 repeats in the database sequence. Primers were chosen with the program Primer 3 (http://frodo.wi.mit.edu/cgi-bin/primer3/primer3_www.cgi (accessed April 2002)); all forward primers were labelled with one of three fluorescent dyes (FAM, HEX, TET). The microsatellites were arranged in two different panels according to magnitude and labelling of the polymerase chain reaction (PCR) product. PCRs were performed as previously described.^18^ For eight microsatellites, we obtained either no PCR product or the corresponding microsatellite was not polymorphic. Therefore 16 microsatellites (supplemental table 1) remained that could be genotyped in 210 trios; these markers cover a genomic region of ∼1.3 Mb around the previously described microsatellites D19S922, D19S865 and D19S916.^17^ PCR products were pooled and size fractionated on an ABI3100 DNA analysis system (Applied Biosystems, Foster City, California, USA) along with a TAMRA labelled standard. Electrophoresis files were analysed with Genotyper and/or Genemapper software (Applied Biosystems). Consistency for correct allele sizes was ensured by simultaneous genotyping of previously defined DNA controls. The overall genotyping rate was 93.3%. Genotypes were checked for Mendelian inheritance, while the transmission disequilibrium test (TDT) as implemented in the family based association test (FBAT) software^22^ was used to test for association under default options.

**Table 1:** Allele frequencies of the associated tag SNP in (A) 1114 psoriasis patients and 937 controls and (B) carriers of the PSORS1 risk allele (633 psoriasis patients and 131 control individuals) and results of χ^2^ statistics

SNP rs-ID	Allele	(A)			(B)			
		Controls	Psoriasis vulgaris	p Value	Controls	Psoriasis vulgaris	p Value	OR (95% CI)
		n (%)	n (%)		n (%)	n (%)		
rs8100377	T	1630 (88.6)	1944 (88.2)	NS	226 (89.7)	1109 (88.6)	NS	NA
	C	210 (11.4)	260 (11.8)		26 (10.3)	143 (11.4)		
rs6511831	A	831 (45.0)	1051 (48.1)	NS	112 (43.8)	594 (48.0)	NS	NA
	G	1015 (55.0)	1135 (51.9)		144 (56.3)	644 (52.0)		
rs8109594	G	1470 (79.5)	1714 (81.4)	NS	206 (79.8)	964 (80.2)	NS	NA
	A	378 (20.5)	392 (18.6)		52 (20.2)	238 (19.8)		
rs12459358	G	1114 (61.1)	1368 (62.8)	NS	141 (55.1)	793 (64.4)	0.005	1.47 (1.12 to 1.94)
	A	708 (38.9)	810 (37.2)		115 (44.9)	439 (35.6)		
rs8102472	T	537 (30.9)	700 (33.4)	NS	63 (26.0)	405 (34.2)	0.013	1.48 (1.08 to 2.02)
	C	1199 (69.1)	1398 (66.6)		179 (74.0)	779 (65.8)		
rs7249334	A	652 (35.2)	779 (37.0)	NS	80 (30.8)	461 (38.2)	0.027	1.39 (1.04 to 1.85)
	G	1198 (64.8)	1325 (63.0)		180 (69.2)	747 (61.8)		
rs10413384	G	1458 (79.1)	1732 (78.4)	NS	198 (76.2)	992 (78.9)	NS	NA
	A	386 (20.9)	478 (21.6)		62 (23.8)	266 (21.1)		
rs2591618	C	1080 (58.1)	1307 (58.9)	NS	144 (55.0)	759 (60.2)	NS	NA
	T	778 (41.9)	913 (41.1)		118 (45.0)	501 (39.8)		
rs28699225	G	1461 (79.7)	1739 (79.0)	NS	194 (77.6)	996 (79.9)	NS	NA
	A	371 (20.3)	461 (21.0)		56 (22.4)	250 (20.1)		

CI, confidence interval; NA, not applicable; NS, not significant; OR odds ratio; SNP, single nucleotide polymorphism.

### Sequencing, LD structure and SNP genotyping

In the genomic region of D19SPS20 and D19SPS21, about 225 kb of sequence were analysed for conservation with mouse sequence and the sequences of other species (as identified with the feature of University of California Santa Cruz (UCSC) genome browser at http://genome.ucsc.edu/ (accessed November 2005)). Those sequences exhibiting evidence for conservation were selected; primers for coverage of these sequences were chosen with Primer 3. In 32 independent psoriasis patients chosen from affected individuals of the trios, 105 PCR products were amplified and sequenced as previously described.^9^ In order to uncover the LD structure of this region, we filtered for single nucleotide polymorphisms (SNPs) with a minor allele frequency (MAF) of >0.10.

Based on the LD structure, we chose a first set of 44 haplotype tagging SNPs (htSNPs) and genotyped them as TaqMan assays in the 300 trios. After first evidence for association of psoriasis to several single SNPs, we chose 24 independent individuals of this study group that were either homozygous (n = 19) or heterozygous (n = 5) for a haplotype consisting of several associated SNP alleles within the LD block. We sequenced the probands for a further 29 PCR products within/neighbouring this LD block to identify further possible risk variants. In the meantime the first HapMap data had been made publicly available. We combined information from both sources to choose an appropriate and redundant set of 30 further htSNPs with different programs such as SNPtagger,^23^ Ldmax^24^ and Haploview.^25^ These SNPs were also genotyped in 300 trios with two different methods—SNPlex or TaqMan (Applied Biosystems)—as recommended by the manufacturer.

For data analysis, only SNPs with a MAF >5%, no deviation from Hardy–Weinberg equilibrium (HWE) (p values >0.01) and no excess of Mendelian errors were accepted. For the remaining 63 SNPs (supplemental table 2) overall genotyping rate was 96.0%. For two SNPs that were genotyped with both methods (SNPlex and TaqMan) the concordance rate was >99.5%. For each SNP a subset of genotypes was confirmed through direct sequencing in 24–32 randomly chosen probands. Genotyping of the same DNA of a control individual was part of every single experiment and resulted in consistent genotypes for all SNPs included in this study.

**Table 2:** Allele frequencies of the risk haplotype in (A) 1114 psoriasis patients and 937 controls and (B) carriers of the PSORS1 risk allele (633 psoriasis patients and 131 control individuals) and results of χ^2^ statistics

Haplotype(s)	Controls	Psoriasis vulgaris	p Value	OR (95% CI)
	n (%)	n (%)		
(A)				
TAGGTAGCG	552.6 (29.5)	698.7 (31.4)	NS	NA
Σ non-risk	1320.6 (70.5)	1526.5 (68.6)		
(B)				
TAGGTAGCG	64.7 (24.7)	403 (31.8)	0.012	1.42 (1.05 to 1.93)
Σ non-risk	197.2 (75.3)	864.3 (68.2)		

CI, confidence interval; NA, not applicable; NS, not significant; OR, odds ratio.

The case control study—1114 PsV patients and 937 control probands—were genotyped for nine SNPs in the associated LD block (rs8100377, rs6511831, rs12459358, rs8109594, rs8102472, rs7249334, rs10413384, rs2591618 and rs28699225) either with SNPlex or TaqMan. Similarly, no SNP showed significant deviation from HWE. Overall genotyping rate was 97.6%. SNPs of two genes of the IL-23R pathway, namely *IL12B* and *IL23R*, were genotyped as recently described.^20^

### LD analysis, statistical and haplotype analysis

LD of tagging SNPs was determined with the software Haploview.^25^ LD blocks were basically defined according to the model “Solid Spine”. In order to be able to include several sibs of one family, we used the TDT as implemented in the association method for single SNPs and haplotypes as described in Becker and Knapp.^26^ This method can be viewed as a generalisation of the TDT.^27^ Haplotypes were calculated within the predefined LD blocks. In the case–control study, association with single SNPs was tested with Armitage’s trend test.^28^ Odds ratios (ORs) and their confidence intervals were determined for single SNP alleles with final p values of <0.05. Haplotypes within the blocks were calculated with the same method as in trios.^26^

In order to compare results from the family study (TDT statistics) with those of case–control studies, we used the model of Kazeem and Farrall^29^ and estimated ORs for SNP rs1249358 from the TDT data and calculated a combined OR of both studies, family based and case–control.

### Stratification for PSORS1 and interaction with other psoriasis susceptibility alleles

For stratification for the PSORS1 risk allele, we used an estimate as previously described.^20^ In order to test for interaction in the case–control study between known psoriasis susceptibility loci and the risk alleles identified at PSORS6, we used logistic regression as suggested by Cordell and Clayton.^30^ We compared a model of three degrees of freedom with an interaction parameter and one parameter for each, the PSORS1 risk haplotype and the SNP rs1245938 at PSORS6, to a model of two degrees of freedom with just the parameters for the markers. In the nuclear families, we compared the number of transmissions/non-transmissions of rs1245938 at PSORS6 after stratification of index cases for carrying the PSORS1 risk haplotype.

In order to test for possible interaction of susceptibility factors of the IL-23R pathway and the PSORS6 variant rs1249358 in the family cohort, we used the variant of IL-23R pathway that was most highly associated in the trios (rs6887695) and tested for interaction with the program UNPHASED.^31^

### Correction for multiple testing

In the family based cohort, we performed an explorative study with microsatellites with few alleles (mostly two to three frequent ones) in order to exclude/confirm a role of PSORS6 in psoriasis. Due to the explorative character of this study, we did not correct for the number of markers/alleles tested. In order to correct for the number of SNPs tested in the second association study, also accounting for LD, we applied a Monte Carlo simulation procedure for the most strongly associated SNP.^26^ Taking into account that we considered the complete sample as well as the samples stratified according to PSORS1 and the *IL12B* variant, we applied a further Bonferroni correction by a factor of 3. To account for the fact that we used different methods to do analysis stratified for PSORS1 on the one hand and for *IL12B* on the other hand (also we did apply only one kind of analysis in each situation), we applied a further Bonferroni correction by a factor of 2.

In order to find out whether the strongly associated SNPs might be the disease-causing variant(s) or whether there is evidence for other not yet identified variants, we performed haplotype analyses. This analysis again was regarded as explorative.

### In silico analyses

We performed in silico analyses to identify potential unknown genes within the associated region. Several gene prediction programs were used: GrailEXP, Genscan and Augustus (32 33 http://augustus.gobics.de/ (accessed November 2005)). Furthermore, we analysed human expressed sequence tags (ESTs) within this interval that were annotated at the UCSC genome browser (http://genome.ucsc.edu/ (accessed November 2005)) and performed blast analysis (http://www.ncbi.nlm.nih.gov/blast/Blast.cgi (accessed November 2005)) to confirm their location within this region. Human and mouse sequences were compared with the Pipmaker software.^34^ In addition, we used two further features annotated in the UCSC genome browser: “mouse chain alignments” and “mouse alignment net”.^35^ Finally, the regions that showed evolutionary conservation were screened for protein sequences with protein blast (blastx, http://www.ncbi.nlm.nih.gov/blast/Blast.cgi (accessed November 2005)).

### Transcription analyses

Genomic intervals that contained in silico gene predictions and showed evolutionary conservation were considered for reverse transcriptase PCR (RT-PCR) based transcription screening. We used a set of primers within these intervals and performed systematic RT-PCRs from cDNA of four different tissues relevant for psoriasis and/or other autoimmune diseases: two cDNAs from skin, blood leucocytes and thymus, respectively, and one from thyroid. Furthermore, we chose forward primers for different RT-PCRs covering the 5′ region of a differential transcript predicted by Genscan. This transcript was longer in the 5′ region than the longest annotated mRNA of *MUC16*, and reached into our associated genomic interval. In combination with reverse primers within the 5′ region of the mRNA of *MUC16*, we performed again exon overlapping RT-PCRs. Moreover, exon-overlapping primers derived from 5′ exons of the *MUC16* RefSeq sequence were selected.

One positive (*GAPDH*) and one negative control (an intronic fragment of *PTPN22*) were included in each experiment. RT-PCRs were performed with invitrogen taq polymerase (Invitrogen, Carlsbad, California, USA) under standard conditions with a touch-down PCR program; each experiment was performed with or without betain. Successfully amplified PCR products of the expected size were sequenced.

### Analysis of copy number variation using quantitative PCR

In order to analyse the genomic region of BAC RP11-79F15, a bacterial artificial chromosome (BAC) that was previously reported to show copy number variation (CNV; Database of Genomic Variation,^36^), we developed two different quantitative PCRs (qPCR), one within the gene *MBD3L1*, the other one within the non-repetitive 5′ part of *MUC16* gene. Genomic copies of the target regions were compared to the housekeeping gene albumin. qPCRs were set up as previously described.^37^ Sequences of primers and probes are available upon request. Ninety-four psoriasis patients and 94 control individuals were tested for CNV at *MBD3L1*, and 94 control individuals for CNV at *MUC16*. We obtained reliable genotypes (defined as SD <0.15) in 97.9% or 98.9% of individuals, respectively.

### Immunohistochemistry

Immunohistochemical analyses were performed with antibody OC125 (MUC16) purchased from Dako (Carpinteria, California, USA) according to the modified ABC method (avidin biotin peroxidase complex) as recommended by the manufacturer. Twelve skin biopsies—10 of psoriasis vulgaris patients and two of control probands—were analysed.

## Results

**Figure 1:**
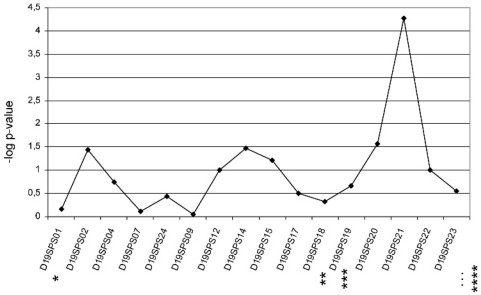
Results of family based association test (FBAT) statistics for 16 microsatellites. Line with rhombi represents results. Stars indicate relative localisation of previously analysed microsatellites (Lee *et al*^17^; *D19S922, **D19S916, ***D19S865 and ****D19S221).

Ten out of 16 microsatellites had two frequent alleles (>5%), a further four markers had three, and two microsatellites had four or five frequent alleles, respectively. FBAT statistics showed strong association with one allele of D19SPS21 in the 210 trios (p = 5.3×10^−5^, fig 1). Of the neighbouring microsatellites, D19SPS20 was less strongly associated (p<2.7×10^−2^).

Sequencing of the 32 individuals in an interval of about 225 kb surrounding D19SPS20 and D19SPS21 identified 87 frequent SNPs with a MAF of >0.10, corresponding to a density of one SNP every 2.6 kb. Haploview identified nine larger LD blocks (supplemental fig 1). One area located between blocks IV and V exhibited remarkably reduced LD and also a lower SNP density. To cover this region better we sequenced additional PCR fragments in a further set of 24 independent PsV patients, either homozygous or heterozygous for the associated haplotype (see below) and combined these data with that from HapMap published in the meantime. This resulted in a dense coverage of 63 successfully genotyped haplotype tagging SNPs, one every 3.3 kb.

**Figure 2:**
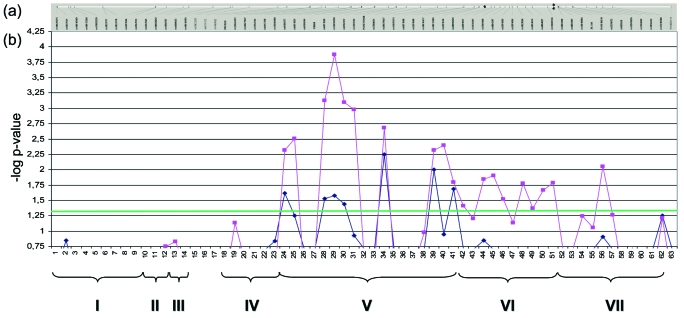
Results of transmission disequilibrium test (TDT) statistics for the 63 haplotype tagging single nucleotide polymorphisms (SNPs). (a) Relative locations of SNPs between bp 8904347 and 9112258 on chromosome 19 (hg18). Stars indicate the location of the two associated microsatellites (*D19SPS20, **D19SPS21). (b) The blue line displays negative logarithms of p values <0.2 in all trios, the magenta line the ones in trios with index patients carrying the PSORS1 risk allele. The green line indicates the significance level of p<0.05, curly braces affiliation to a haplotype block as shown in fig 3 and supplemental table 2.

**Figure 3:**
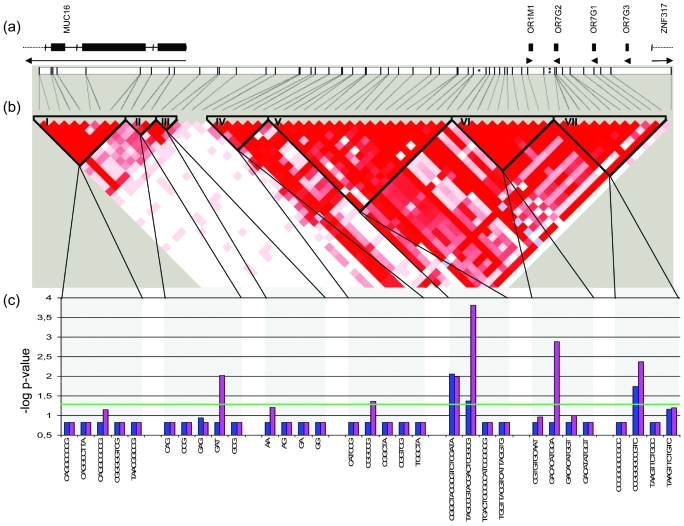
Genomic region of interest, linkage disequilibrium (LD) structure in trios, and results of transmission disequilibrium test (TDT) statistics for haplotypes within seven haplotype blocks. (a) Name, exon structure and orientation of RefSeq genes. (b) Pairwise LD plot for the 63 single nucleotide polymorphisms (SNPs) based on data of trios with index patients carrying the PSORS1 risk allele. Each square plots the level of LD between a pair of sites in the region; comparisons between neighbouring sites lie along the upper most line. Red colouring indicates strong LD, light red less strong LD, a shadow of red intermediate LD, and white indicates weak LD. The areas limited by a black line show the seven haplotype blocks basically defined by the model “Solid Spine”. (c) Results of FAMHAP statistics for haplotypes with >30 informative transmissions in all trios; shown are results within the seven haplotype blocks in all trios (blue) and in trios with index patients carrying the PSORS1 risk allele (magenta). The green line indicates the significance level of p<0.05, stars the location of the two associated microsatellites (*D19SPS20, **D19SPS21). For p values >0.15, a negative logarithm of 0.15 is shown.

In the 300 trios, association to several single SNPs in the centre of the interval—SNPs 24 to 41, corresponding to block V—was the main finding (fig 2, supplemental table 2). Also at the haplotype level, association was observed in blocks IV to VI (fig 3). However, in comparison with findings in the microsatellite scan, evidence for association was less significant (p<0.006). After stratification of index patients of all trios to the PSORS1 risk allele, association findings were substantially stronger in the trios with index patients carrying the PSORS1 risk allele ( =  PSORS1 positive trios; fig 2 and 3), while no evidence for association was identified in PSORS1 negative trios. Four single SNPs in LD block V (rs6511838, rs12459358, rs8102472, rs7249334) gave the strongest association signal; the corresponding transmission ratios were 88:46, 92:47, 80:41 and 80:42 (T:U), respectively (p values between 1.0×10^−4^ and 8.0×10^−4^ (fig 2)). Similarly, a frequent haplotype (32.0%) within this LD block, which was a combination of mostly associated alleles (TAGCCGTACGACTCGGCG), showed a similarly skewed transmission ratio of 37:12 (p = 1.0×10^−4^ (fig 3)). None of the further variants of this haplotype identified through sequencing showed association indicating that (one of) the four variants or a yet unidentified one(s) that are in strong LD with them are disease-causing. After correction for the number of SNPs tested, the best single marker association p value of p = 0.000132 for rs12459358 increased to 0.0046. The further Bonferroni correction in order to correct for the different stratification strategies resulted in a p value of 0.0276 in the family sample.

In order to replicate our association finding, we analysed an independent case–control study of 2051 individuals for nine single SNPs within LD block V. Initially, we did not observe evidence for association of PsV to any of the single SNPs or the corresponding haplotypes (tables 1 and 2). After stratification to the PSORS1 risk allele, though, three SNPs (rs12459358, rs8102472 and rs7249334) showed large allele frequency differences of about 8% resulting in significant p values of 5.0×10^−3^, 0.013 and 0.027 in carriers of the PSORS1 risk allele (table 1). The corresponding haplotype TAGGTAGCG within block V had a frequency of 31.8% in these latter patients and of 24.7% in the corresponding control individuals (p = 0.012; OR 1.42, 95% CI 1.05–1.93) (table 2). Since the case–control study was regarded as a replication study, we initially did not correct for the number of tests performed. However, the best p value of 0.005 (for rs12459358) would withstand MC based correction for the number of SNPs tested (p = 0.034; same method as for the family sample) as well as Bonferroni correction (p = 0.045).

We chose SNP rs12459358 to compare association effects of both studies by the model of Kazeem and Farrall.^29^ While the OR for rs12459358 in the subset of PSORS1 positive trios was estimated to be 2.6±0.20 and therefore higher than the one of the case–control study, the combined OR was estimated to be 1.78±0.11. Although we detected evidence for significance of this latter value (χ^2^ = 25.37, p<4.7×10^−7^), test of homogeneity of ORs indicated significant heterogeneity.

Our interaction analysis in the case–control study with regard to risk alleles of PSORS1 and PSORS6 revealed that the interaction parameter significantly improved the model fit (p = 0.044). We have thus significant deviation from a multiplicative two marker model. In addition, the skewed transmission ratio in the independent family based cohort was even more significant (p = 1.65×10^−3^), indicating evidence for interaction in both, the family and the case–control groups. In contrast, no evidence for interaction between the PSORS6 risk allele and variant rs6887695 of the *IL12B* gene was detected (p = 0.41).

The analyses with gene prediction programs did not provide interesting candidate genes/regions. Genscan predicted six hypothetical genes plus additional 5′ exons of the annotated gene *MUC16*. These six gene suggestions were unlikely to be real since almost none of the exons overlapped evolutionary conserved regions. Further in silico analyses of annotated ESTs showed that only 13 of the 44 ESTs revealed a satisfying alignment with the region of interest and these mostly overlapped with one of the many repetitive elements.

**Figure 4:**
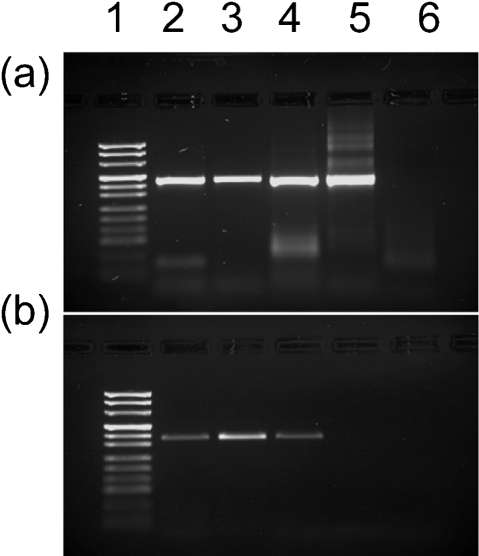
Electrophoresis of conventional reverse transcriptase polymerase chain reaction (RT-PCR) products demonstrating expected amplicon sizes of a product for each (a) GAPDH and (b) *MUC16* (primers in exons 4+5, PCR product of 363 bp), amplified from cDNAs of different tissues: lane 1: size standard, 2: skin, 3: thymus, 4: thyroid, 5: blood leucocytes, 6: non-template control.

**Figure 5:**
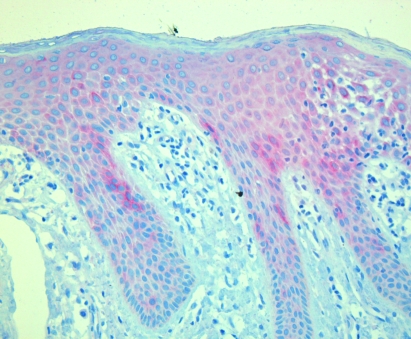
Typical histology of psoriasis vulgaris with elongated ridges of epidermis and strong inflammatory infiltrate in the papillary dermis. Note MUC16 staining, although faintly present in basal epidermal cells, shows strongest expression in suprabasal cells of the stratum spinosum, in particular the suprapapillary epidermis, while expression in rete ridges and in the granular layer of the epidermis is less pronounced.

Alignment of corresponding human and mouse genomic sequence showed 20 regions of good conservation with similarities to known genes from unlinked regions. Nevertheless, we were unable to amplify any RT-PCR products for any of the regions from skin, thyroid, thymus and/or blood leucocytes. Also the predicted additional 5′ exons of the annotated *MUC16* mRNA were undetectable in cDNA from these tissues. In contrast, we detected transcription of *MUC16* mRNA in all tissues but leucocytes (fig 4). These results were confirmed in independent experiments using different primers (data not shown). Accordingly, immunohistochemical analyses using an antibody against MUC16 showed immunostaining in epidermal cells of all 12 skin biopsies investigated. In addition, several individuals—four patients and one control individual—showed focally more intensive staining in several areas of basal and suprabasal epidermal cells (fig 5).

Finally, we were able to develop two robust assays for detecting variation in copy number in the region of BAC RP11-79F15, overlapping the 3′ region of *MUC16* telomeric to the associated region. This region was previously reported to be polymorphic for copy number in the European population. However, we did not find any loss or gain of copy number in any of the analysed 184 and 93 individuals, respectively. Additional data from Affymetrix 6.0 SNP chips of 158 individuals analysed with the Affymetrix Console 2.1 did not show CNV of this region (data not shown). We conclude that the region of this BAC shows no significant variation in copy number, at least in the German population.

## Discussion

The adaptation of the classical linkage analysis paradigm, initially developed for gene identification of Mendelian disorders, to complex traits is based on an initial genome-wide linkage scan followed by LD studies in regions showing evidence for linkage. This strategy has been successful for several diseases such as Crohn’s,^3^ ^38^ asthma^39^ and sarcoidosis^40^ to name a few. We followed this strategy with an initial genome-wide linkage scan of 32 extended PsV families with multiple affected individuals. This study identified two major linked regions, one at the HLA locus on chromosome 6 (PSORS1) and one on chromosome 19p13 (PSORS6) (Lee *et al*^17^). PSORS1 is the major PsV susceptibility locus and has been replicated in all linkage studies to date, while the PSORS6 has only been replicated in a study from the UK.^41^

We performed a systematic LD study in the region of the linkage peak at psoriasis susceptibility locus 6 (PSORS6). When scanning a 1.3 Mb region from the core linkage interval using 16 informative microsatellites with reduced heterozygosity, we were able to identify a strong association to one allele of microsatellite D19SPS21. Since the neighbouring marker D19SPS20 was also associated, we focused our efforts on this region of about 250 kb. At the time of discovery, the LD structure of this region was poorly characterised. We therefore identified SNPs in 32 patients, determined the LD structure, and confirmed it by genotyping tagging SNPs in 600 independent parents of the trio cohort. The structure proved to be concordant with that later reported by HapMap.^42^ In the TDT analysis in 300 trios we found association with several SNPs from the same 50 kb haplotype block. Similarly, the haplotype encompassing the same alleles was also associated. The association signals increased after stratification for the PSORS1 risk allele on chromosome 6p, suggesting that both risk factors interact.

To verify these findings we genotyped tagging SNPs for this haplotype in an independent case control cohort of 1114 patients and 937 controls. After stratification for PSORS1 the association was again significant, although less strongly. Nevertheless, we consider this a successful replication. While we found evidence for interaction with the PSORS1 risk allele, a similar effect of variants of the IL-23R pathway was not detectable. The combined OR of both study groups for one SNP at PSORS6 was estimated to be 1.78±0.11. This magnitude of susceptibility is in the range observed in non-HLA loci in psoriasis and many other complex diseases. For example, the recently described and well replicated variants in genes of the IL-23R pathway^15^ ^16^ showed a similar effect size in our German case control cohort (OR 1.50).^20^

Interaction between PSORS1 and one of the further psoriasis susceptibility loci has been described previously for PSORS4 on chromosome 1q21.^43^ In our study, stratification according to PSORS1 indicated that PSORS6 was only relevant in PSORS1 carriers. Indeed, we did not observe association of psoriasis with POSRS6 in non-carriers of the PSORS1 risk allele. Thus, we can speak of epistasis, and our model is neither multiplicative nor additive. Our family based sample contained some families with more than one affected child. For this configuration, a derivation of the exact disease model remains a challenge.

Although chromosome 19 has about twice the gene density of the genome average,^44^ our results did not map to any known coding region. Many non-coding regions, though, harbour regulatory elements regulating expression of adjacent genes. Interestingly, we showed that one of the neighbouring genes, mucin 16 (*MUC16*), of unknown function is expressed in several tissues that are relevant for psoriasis and/or other autoimmune diseases such as skin, thymus, and thyroid. Expression in other epithelia such as ocular surfaces (cornea, conjunctiva), respiratory tract and vagina has been described.^45^ ^46^ The highly glycosylated MUC16 has long been known as a tumour marker indicating recurrence of ovarian cancer and preceding cancer genesis^47^ and is often referred to as antigen CA125.^48^ Our immunohistochemical analysis revealed weak general staining of the epidermis with focally increased signals in suprabasal cells of the suprapapillary epidermis. This location coincides with the main hyperproliferative zone in psoriatic skin, while in normal epidermis proliferation is restricted to basal cells.^49^ Our results show a discrepancy with previously performed microarray analyses that did not show differential expression.^50^ ^51^ ^52^ This might be explained by a restricted expression of *MUC16* in the suprabasal layer.

Interestingly, retinoids and glucocorticoids, which are effective therapeutic agents in psoriasis, enhance *MUC16* expression in eye epithelia.^53^ ^54^ ^55^ Furthermore, a role of *MUC16* in protection against pathogen adherence^56^ renders it an even more interesting gene for psoriasis with regard to the *HLA-C* risk allele. Especially PSORS1 positive patients are prone to develop the guttate form of psoriasis,^57^ a form that has been strongly associated with throat infections with β-haemolytic streptococci.^58^ Recently a variant in the 5′ region of another member of the mucin family, *MUC19*, has been newly identified to be relevant in the pathogenesis of Crohn’s disease.^59^ This might indicate a more general role of these epithelial proteins in the pathogenesis of diseases with compromised barrier function. Nevertheless, we cannot exclude that expression of other genes in this genomic region might also be affected. To analyse this aspect exhaustingly, a combination of several functional analyses such as qPCR, expression studies of further positional candidates, for example, would be necessary.

Besides SNPs, copy number variation (CNV) has been recognised as a new class of potential risk factors in complex diseases. In psoriasis, a CNV comprising the β-defensin gene cluster was reported as a risk factor in various European cohorts, including our own study group.^60^ Iafrate *et al*^36^ reported a frequent CNV of the adjacent telomeric region encompassing the 3′ parts of *MUC16* gene as well as *MBD3L1* and *ZNF558* genes. Since LD of the associated SNPs with this CNV could not be excluded, we developed two qPCR assays using an established approach^37^ and genotyped part of the case–control cohort. However, we could not detect this CNV in our population. Furthermore, SNP chip analyses in a further 158 individuals were normal with regard to CNV of this region. Therefore, we can probably exclude a CNV of the adjacent region as an explanation for the association findings. In order to clarify the potential role of *MUC16* in psoriasis and to identify the causal variant(s) at this locus, further functional studies are necessary.

In summary, we identified a susceptibility factor at PSORS6 that is relevant in patients with type I psoriasis carrying the PSORS1 risk allele. This risk factor might modify the expression of neighbouring genes—for example, *MUC16*—in patients carrying this risk factor.
